# Peptidase inhibitor (PI16) impairs bladder cancer metastasis by inhibiting NF-κB activation via disrupting multiple-site ubiquitination of NEMO

**DOI:** 10.1186/s11658-023-00465-6

**Published:** 2023-07-31

**Authors:** Xiangqin Kuang, Zhuojun Zhang, Difeng Li, Wenhao Bao, Jinyuan Pan, Ping Zhou, Han Chen, Zhiqing Gao, Xiaoyi Xie, Chunxiao Yang, Ge Zhu, Zhongqiu Zhou, Ruiming Tang, Zhengfu Feng, Lihuan Zhou, Xiaoli Feng, Lan Wang, Jianan Yang, Lili Jiang

**Affiliations:** 1 Affiliated Cancer Hospital & Institute of Guangzhou Medical University, Guangzhou, 510095 China; 2 Guangzhou Municipal and Guangdong Provincial Key Laboratory of Protein Modification and Degradation, School of Basic Medical Science, Guangzhou Medical University, Guangzhou, 511436 China; 3 Department of Medical Imaging, Health Science Center, Hubei Minzu University, Enshi, 445000 China; 4 Department of Oncology, Huanggang Central Hospital of Yangtze University, Huanggang, 438000 China; 5 Meishan Women and Children’s Hospital, Alliance Hospital of West China Second University Hospital, Sichuan University, Meishan, 620000 China; 6 The Sixth Affiliated Hospital of Guangzhou Medical University, Qingyuan People’s Hospital, Guangzhou, 511518 China; 7 Department of Pathogen Biology and Immunology, School of Basic Courses, Guangdong Pharmaceutical University, Guangzhou, 510006 China; 8 Department of Urologic Oncosurgery, Affiliated Cancer Hospital & Institute of Guangzhou Medical University, Guangzhou, 510095 China; 9 Department of Pathology, School of Medicine, Women’s Hospital, Zhejiang University, 310006 Hangzhou, China

**Keywords:** Bladder cancer, PI16, NF-κB, Ubiquitination

## Abstract

**Background:**

Bladder cancer (BLCA) is a malignancy that frequently metastasizes and leads to poor patient prognosis. It is essential to understand the molecular mechanisms underlying the progression and metastasis of BLCA and identify potential biomarkers.

**Methods:**

The expression of peptidase inhibitor 16 (PI16) was analysed using quantitative PCR, immunoblotting and immunohistochemistry assays. The functional roles of PI16 were evaluated using wound healing, transwell, and human umbilical vein endothelial cell tube formation assays, as well as in vivo tumour models. The effects of PI16 on nuclear factor κB (NF-κB) signalling activation were examined using luciferase reporter gene systems, immunoblotting and immunofluorescence assays. Co-immunoprecipitation was used to investigate the interaction of PI16 with annexin-A1 (ANXA1) and NEMO.

**Results:**

PI16 expression was downregulated in bladder cancer tissues, and lower PI16 levels correlated with disease progression and poor survival in patients with BLCA. Overexpressing PI16 inhibited BLCA cell growth, motility, invasion and angiogenesis in vitro and in vivo, while silencing PI16 had the opposite effects. Mechanistically, PI16 inhibited the activation of the NF-κB pathway by interacting with ANXA1, which inhibited K63 and M1 ubiquitination of NEMO.

**Conclusions:**

These results indicate that PI16 functions as a tumour suppressor in BLCA by inhibiting tumour growth and metastasis. Additionally, PI16 may serve as a potential biomarker for metastatic BLCA.

**Supplementary Information:**

The online version contains supplementary material available at 10.1186/s11658-023-00465-6.

## Background

Bladder cancer (BLCA) is the most prevalent malignancy arising from the mucosal epithelium of the bladder. It primarily affects men and ranks as the sixth most common cancer and the ninth leading cause of cancer-related death among men [1]. Patients with BLCA have a poor prognosis, largely due to the disease’s aggressive progression, local invasion and early metastasis. Approximately 10%–20% of patients initially diagnosed with non-muscle-invasive BLCA eventually progress to the muscle-invasive subtype, which severely limits the available treatment options and results in dismal outcomes [2–4]. Consequently, there is an urgent need to identify key molecules responsible for metastatic BLCA to improve early diagnosis and therapy.

Peptidase inhibitor 16 (PI16), a member of the cysteine-rich secretory proteins, antigen 5, and pathogenesis-related 1 proteins (CAP) superfamily, is a cysteine-rich protein that is widely expressed in human tissues [5]. Studies have shown that PI16 is involved in extracellular matrix regulation, acts as a tumour suppressor gene and is a new independent prognostic marker of prostate cancer [5–7]. However, the role of PI16 in BLCA progression remains unknown. Nuclear factor κB (NF-κB) is a convergence point for multiple metabolic and oncogenic signalling pathways and plays an important role not only in regulating immune responses and inflammation, but also in tumour progression [8–10]. Although a growing body of research indicates that NF-κB signalling activation plays a crucial role in the development of BLCA [11, 12], the regulatory mechanism of this pathway in BLCA progression remains incompletely understood.

This study aimed to investigate the inhibitory effects of PI16 on BLCA progression and metastasis and to elucidate the underlying mechanism of PI16 in BLCA metastasis. Our findings revealed that PI16 expression was abnormally downregulated in BLCA, indicating early disease progression and poor survival in patients with BLCA. Furthermore, PI16 inhibited K63 ubiquitination (K63-Ub) and M1 ubiquitination (M1-Ub) of NEMO by interacting with annexin-A1 (ANXA1), thereby inhibiting the activation of the NF-κB pathway and the subsequent invasion and metastasis of BLCA cells. In summary, our findings provide a basis for using PI16 as a potential diagnostic biomarker and therapeutic target for patients with BLCA.

## Methods

### Cell culture and agents

The BLCA cell lines RT4 (#HTB-2), SCaBER (#HTB-3), UMUC-3 (#CRL-1749), T24 (#HTB-4), J82 (#HTB-1), 5637 (#HTB-9), TCCSUP (#HTB-5) and SW780 (#CRL-2169) and the human umbilical vein endothelial cell (HUVEC) (#PCS-100-010) were purchased from the American Type Culture Collection (ATCC). The BLCA cell lines were cultured in Dulbecco’s Modified Eagle’s Medium (DMEM) (Gibco, Grand Island, NY, USA, #C11995500BT) supplemented with 10% fetal bovine serum (FBS) (Gibco, #0270-106). The HUVEC cell was cultured in endothelial cell medium (Sciencell, Carlsbad, CA, USA, #1001) supplemented with 10% FBS. The immortalized human bladder epithelial cell SV-HUC-1 (#ZQ0345) and the murine BLCA cell line MB49 (#JNO-886) were purchased from Guangzhou Xiandu Biotechnology Company (Guangzhou, CHN). All the cells were cultured in a humidified incubator at 37 °C and 5% CO_2_. The linear ubiquitin assembly complex (LUBAC) inhibitor HOIPIN-8 (400 nM) was purchased from AxonMedchem (Groningen, Netherlands, #2927) and dissolved in dimethyl sulfoxide (DMSO). NF-κB inhibitors JSH-23 (#S7351) and QNZ (#S4902) were purchased from Selleck (Houston, TX, USA) and dissolved in DMSO.

### Tissue specimens and immunohistochemistry (IHC) assay

A tissue array (No. HBlaU108Su01, Outdo Biotech, Shanghai, CHN) was used to detect PI16 expression. Clinical and pathological classification and clinical staging were determined according to the standards of the American Joint Committee on Cancer. The investigation was conducted in accordance with the Declaration of Helsinki and the ethical standards of national and international standards. For the use of clinical material used, the Institutional Research Ethics Committee ethics approval was obtained (2021-09-18). IHC assay was performed according to the previous report [13]. Sections were then incubated with anti-PI16 (Proteintech, #12267-1-AP), p65 (Proteintech, #66535-1-Ig), MMP9 (Proteintech, #10375-2-AP) and CD31 (Proteintech, #11265-1-AP) antibodies. IHC results were quantified using the staining index (SI) and the mean optical density (MOD) determined by ImageJ software [13]. The clinical information of the patients’ samples is shown in Additional file 1: Tables S1, S2.

### RNA extraction, reverse transcription, and quantitative real-time PCR

Total RNA was extracted from the cultured cells using Trizol reagent (Invitrogen, Carlsbad, CA, USA, #15596018), RNA was quantified using NanoDrop ND-1000 and PrimeScript™ RT Master Mix (Takara Biomedical Technology, Beijing, #RR036A) was used according to the manufacturer’s instructions to perform reverse transcription. Quantitative real-time PCR (qPCR) was performed on an Applied Biosystems 7500 Fast Real-Time PCR System using TB Green^®^ Advantage^®^ qPCR Premix (Takara, #639676). GAPDH was used as an endogenous control and the relative expression levels were calculated using the 2^−ΔΔCT^ method. The information on primers is shown in Additional file 1: Table S3.

### Plasmids, shRNA and transfection

pLent-PI16 was generated by cloning the PCR-amplified human PI16 coding sequence (NM_153370) into the pLent-vector (DHbio, Guangzhou, CHN). BLCA cells were transduced with lentiviral particles expressing PI16 or short hairpin RNA (shRNA) (Vigene Biosciences, Shandong, CHN) targeting the PI16 sequence, facilitated by Polybrene, according to the manufacturer’s instructions. A total of 48 h after virus infection, cells stably expressing PI16, or those silenced for 10 days, were selected with 0.5 μg/mL puromycin (Selleck, #S9631). The expression of PI16 was confirmed by western blot analysis.

### In vivo tumour models

All animal experimental procedures were approved by the Institutional Animal Care and Use Committee of Guangzhou Medical University (SQ2021-017/2021-9-18) and carried out in accordance with the Basel Declaration and the National Research Council’s Guide for the Care and Use of Laboratory Animals. BALB/c nude mice (male, 4–5 weeks, 18–20 g) were purchased from Beijing Vital River Laboratory Animal Technology (Beijing, CHN, # 801-667). MB49 cells stably expressing PI16-vector (vec), PI16, shRNA-vector (sh-vec) and shRNA-PI16 (sh-PI16) were constructed. BALB/c nude mice were randomly divided into two groups for subcutaneous implantation. One group was seeded with MB49-vector cells (2 × 10^6^) in the left back and MB49-PI16 cells (2 × 10^6^) in the right back. The other group was inoculated with MB49-shRNA-vector cells (2 × 10^6^) in the left back and MB49-shRNA-PI16 cells (2 × 10^6^) in the right back. Tumour growth was monitored every 7 days and photographed and imaged. Mice were anaesthetized and injected intraperitoneally with D-Luciferin (100 μL of 15 mg/mL in D-PBS; PerkinElmer, Waltham, MA, #122799) and images were captured using IVIS^®^ Spectrum In Vivo Imaging System (PerkinElmer). After injection of D-Luciferin for 5 min, mice were anaesthetized by the inhalation of isoflurane with a RAS-4 Rodent Anesthesia System equipped with an Isoflurane vaporizer (PerkinElmer) and exposed to 2% isoflurane delivered in oxygen within a 2.5-L induction chamber. Mice were then placed in the light-tight imaging chamber; anaesthesia was continued during the procedure with 2% isoflurane introduced via a nose cone. The IVIS software was opened, and the charge-coupled device camera was selected to initialize cooling to − 90 °C. Image acquisition settings were as follows: imaging mode auto and exposure time auto. Image analysis and quantification were done using IVIS software; quantification and an ROI for quantification were selected. The results tab displayed the calculated values for each image that was included in the analysis. Tumour volume was measured from two directions with a vernier calliper and calculated as follows: (length × width^2^)/2. Mice were euthanized 30–35 days after tumour transplantation, and tumours were removed and weighed. Tumours were fixed in formalin. For mouse tail vein lung metastasis, the indicated cells (2 × 10^6^) were seeded from the tail vein of nude mice for 1 week after in vivo imaging was performed to observe the lung metastasis; the mice were then humanely euthanized after 30 days, and the lung tissue was extracted and fixed in picric acid.

### Cell proliferation analysis

The indicated cells (1 × 10^3^/well) were seeded into 96-well plates and allowed to adhere and grow for 12–24 h at 37 °C in a humidified CO_2_ incubator. A volume of 20 μL MTS reagent (Promega, Madison, WI, USA, #G3581) was added to each well, including wells with no cells as a blank. Incubate the plate at 37 °C for 2 h. Cell viability was assessed on the basis of the absorbance optical density. Measure the absorbance at 490 nm using a 96-well plate reader. Blank the plate reader using wells containing MTS reagent and no cells.

### Western blot assay

The cells were treated as indicated and were prepared in the RIPA lysis buffer (Cell Signaling Technology, Danvers, MA, USA, #9806) and quantified using a BCA kit (Thermo, Waltham, MA, USA, #23227). The following primary antibodies were used: PI16 (Proteintech, Rosemont, IL, USA, #12267-1-AP), NEMO (#ab178872), HOIL-1 (#ab108479) (Abcam, Cambridge, MA, USA), p-IKKα/β (#2697), IKKα (#11930), IKKβ (#8943), IκBα (#4814), p-IκBα (#2859), p65 (#8242), p-p65 (#4887), GAPDH (#5174), p84 (#131268), K63-ubiquitin (#12930), ANXA1 (#32934), HOIP (#99633), Sharpin (#12541) (Cell Signaling) and M1-ubiquitin (Millipore, #MABS451). The secondary antibody (Cell Signaling, #7074, #7076) was probed at the indicated time points. The immunoreactive strips were detected using the ECL Ultra Western HRP Substrate (Millipore, #WBULS0100) detection kit.

### Transwell matrix penetration assay and transwell assay

Transwell with an 8.0-µm pore polyester membrane insert was purchased from Corning (Corning, Painted Post, NY, USA, #3422); the cells (1 × 10^4^) were seeded on the top chamber of Transwell^®^ Permeable Supports (Corning) and coated with or without the Cultrex Basement Membrane Extract (BME) (R&D Systems, Minneapolis, MN, USA, #3432-010-01). Ligand containing 10% FBS was added to the bottom of the chamber. After 24 h of culture at 37 ℃, transwells were rinsed with phosphate buffered saline (PBS) twice and fixed with 4% paraformaldehyde for 15 min, the cells at the bottom of the upper chamber were gently swabbed, viewed under a microscope, photographed and counted.

### Human umbilical vein endothelial cells tube formation assay

A total of 200 μL of the BME (R and D Systems) was drawn and spread on the bottom of a 24-well plate and incubated at 37 °C for 1 h. HUVECs were trypsinized, counted and resuspended at a concentration of 2 × 10^5^ cells/mL in EGM-2 medium (which contains growth factors required for endothelial cell growth). A total of 200 μL of the cell suspension (40,000 cells) was added to each well of the BME-coated plates, cultured in the indicated conditioned medium and incubated at 37 °C for 6–12 h. We checked for tube formation under a light microscope, looking for elongated and connected cellular structures. Images were taken under the microscope and capillaries were quantified by calculating tube lengths and branch points.

### Luciferase reporter assay

The cells (5 × 10^4^) were seeded in 24-well plates and left to stand for 24 h. NF-κB Firefly luciferase reporter plasmid (500 ng, Promega, Madison, WI, USA, #E8491) and pRL-TK Renilla plasmid (100 ng, Promega, #E8491) were co-transfected into the cells with EZ reagent (Life-iLab, Shanghai, CHN, #ACO4L092). Thirty-six hours after transfection, the luciferase and Renilla signals were measured using the Dual-Luciferase Reporter Assay Kit (Promega, #E1960). After lysing the cells with Passive Lysis Buffer (PLB), an equal volume (100 μL) of Dual-Glo® Luciferase Reagent was added to each well containing the transfected cells. Then, Firefly luminescence in the sample was measured using a plate reader set to detect bioluminescence. Dual-Glo^®^ Stop and Glo^®^ Reagent was added to quench the Firefly reaction and provide the substrate for Renilla luminescence; Renilla luminescence was measured to determine the internal control value. The ratio of Firefly to Renilla luminescence was calculated. This normalized the Firefly values to the internal control, accounting for transfection efficiency. Normalized promoter values were compared between samples to determine changes in promoter activity.

### Statistical analysis

The statistical tests for the analysis of experimental data in this study included Fisher’s exact test, log-rank test, chi-squared test and *t*-test. Survival curves were plotted by the Kaplan–Meier method and compared with the log-rank test. The receiver operating characteristics (ROC) curve analysis was used to determine the diagnostic value of PI16 expression in patients with BLCA. The construction of the ROC curves was performed using GraphPad Prism and the areas under the ROC curve (AUC) with a 95% confidence interval (CI) were calculated to evaluate the diagnostic accuracy and discrimination power. Gene Set Enrichment Analysis (GSEA) analysis was originally used to analyse the theoretical relevance of multiple bio-functions related to PI16 expression [14, 15]. The gene sets of the Molecular Signatures Database (MSigDB) and data from the Cancer Genome Atlas (TCGA) were used for the GSEA analysis. For each sample and signature, GSEA reported a signature expression score between 0 and 1 and the statistical significance (*P* value) for signature overexpression. Statistical analysis was performed using the SPSS 25.0 (IBM SPSS Statistics, IL, USA) statistical package. Data are shown as mean ± standard deviation (SD) values and *P* ≤ 0.05 was considered statistically significant.

## Results

### Downregulation of PI16 in BLCA correlates with poor prognosis in patients

**Fig. 1:**
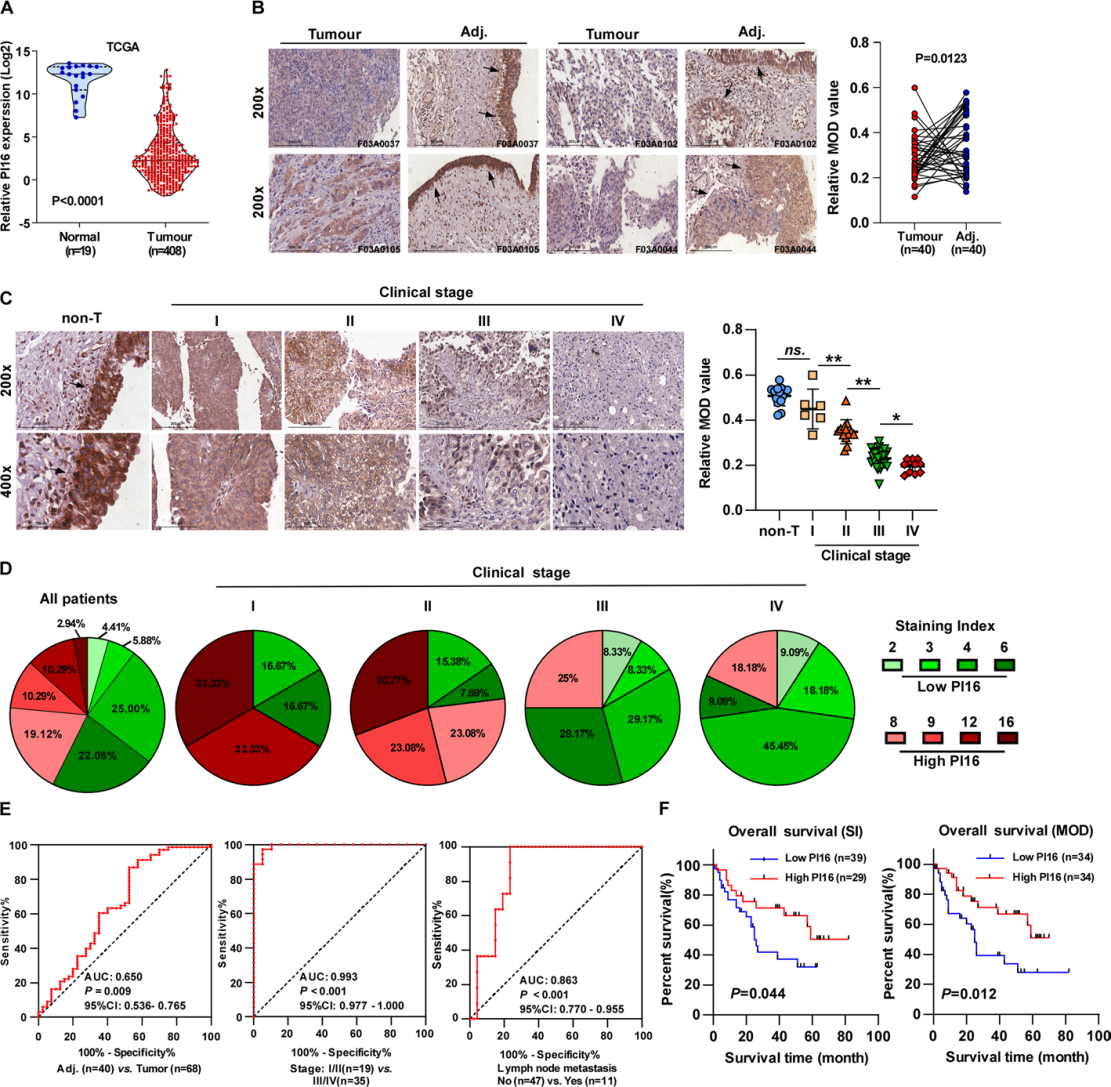
PI16 is downregulated in BLCA and correlates with poor patient survival. **A** TCGA analysis of PI16 mRNA in 19 normal and 408 BLCA tissues (*P* < 0.0001). **B** Representative images of IHC analysis of PI16 in BLCA (tumour) and adjacent normal tissue (adj.). MOD values quantified IHC. **C** Representative images and quantification of IHC analysis of PI16 in non-tumour bladder epithelial tissues (non-T) and stage I–IV BLCA; MOD values quantified IHC, scale bar 200 μm. **D** PI16 staining index (SI) distribution; SI ≥ 8, high expression. **E** ROC analysis of PI16 in the indicated groups. **F** Kaplan–Meier analysis of 68 patients with BLCA; low/high PI16 by SI/MOD cut survival info. Log-rank test calculated *P* value. A two-tailed *t*-test was used for statistical analysis. Error bars represent the mean ± SD of three independent experiments. *P < 0.05; **P < 0.01

To explore the potential role of PI16 in carcinogenesis, we analysed data from the TNMplot database and found PI16 expression was downregulated in 22 human cancers (Additional file 1: Fig. S1A). Analysis of TCGA data showed PI16 expression was significantly downregulated in BLCA tissues compared with normal tissues (Fig. 1A). Furthermore, IHC staining of a tissue array (no. HBlaU108Su01), including 40 pairs of BLCA and adjacent normal tissues, consistently showed PI16 was downregulated in BLCA tissues compared with normal tissues (Fig. 1B). PI16 expression was specific to normal and neoplastic bladder epithelial tissues but undetectable in adjacent stromal tissues (Additional file 1: Fig. S1B). Our results further showed PI16 expression negatively correlated with BLCA clinical stage (Fig. 1C). The percentage of BLCA tissues with high PI16 expression (defined as SI ≥ 8) decreased with increasing clinical stage from I to IV (Fig. 1D).

To evaluate the diagnostic value of PI16 in BLCA, we performed a ROC curve analysis. PI16 expression distinguished BLCA from normal tissues (AUC = 0.650, *P* = 0.009). Strikingly, PI16 better-distinguished early-stage (I/II) from late-stage (III/IV) BLCA (AUC = 0.993, *P* < 0.001). PI16 also indicated lymph node metastasis (AUC = 0.863, *P* < 0.001) (Fig. 1E). These results demonstrate PI16 downregulation has high diagnostic value for determining BLCA progression and metastasis.

Kaplan–Meier analysis of 68 patients with BLCA showed lower PI16 expression correlated with shorter survival (Additional file 1: Table S1; *P* < 0.05, Fig. 1F). Univariate and multivariate analyses identified PI16 expression as an independent prognostic marker (Additional file 1: Table S2). Compared with normal urothelial cells (SV-HUC-1), PI16 expression was downregulated in all eight BLCA cell lines (Additional file 1: Fig. S1C), suggesting PI16 may act as a BLCA suppressor. In summary, downregulated PI16 indicates poor prognosis in patients with BLCA and may serve as a diagnostic and prognostic biomarker for this disease.

### PI16 suppresses tumorigenicity and metastatic progression of BLCA in vivo and in vitro

**Fig. 2:**
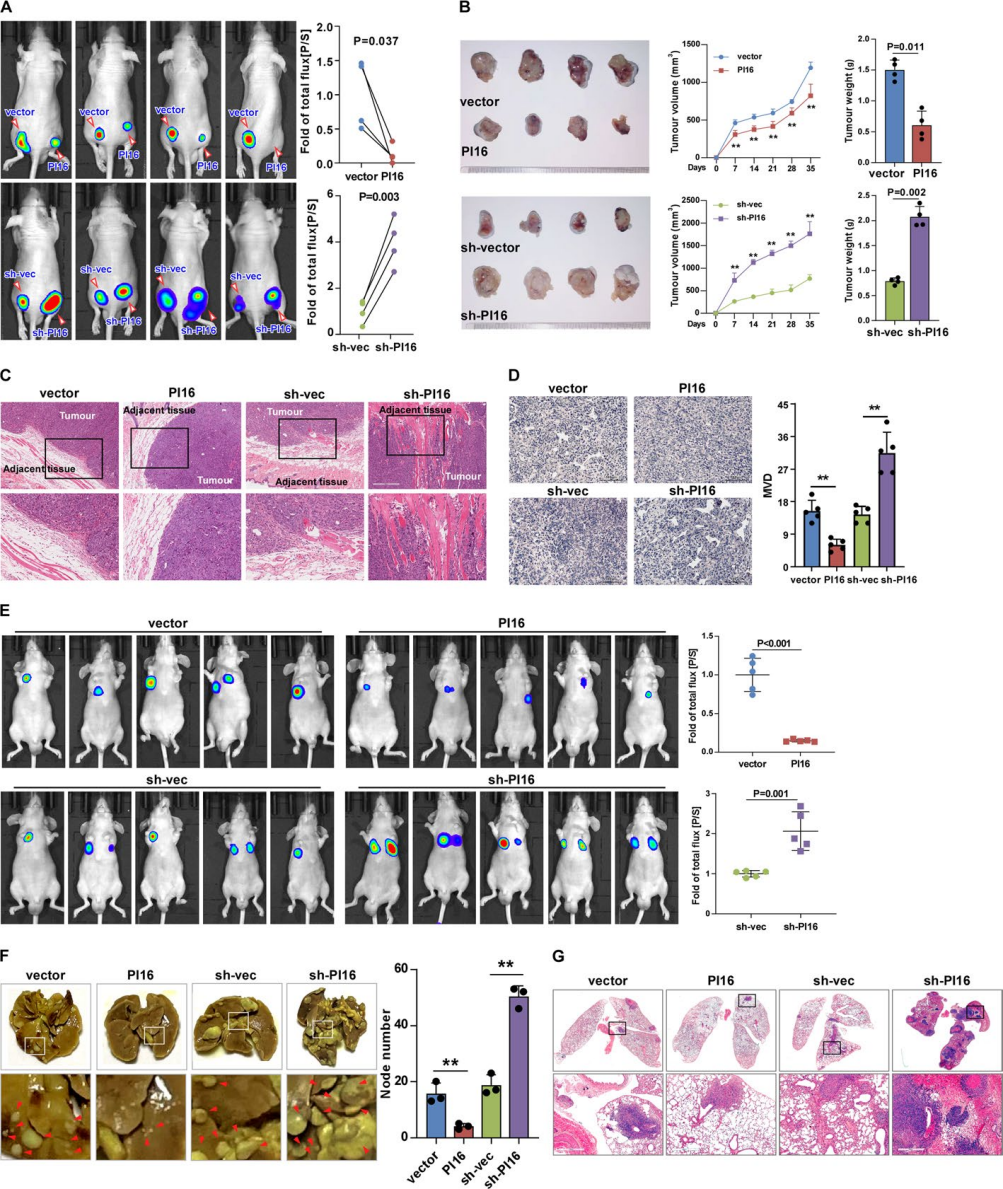
PI16 suppresses BLCA metastasis in vivo. **A** Representative images of in vivo imaging system (IVIS) detection of subcutaneous tumours in nude mice (left) and their quantitation (right). **B** Representative images of subcutaneous tumours; the volume and weight of tumours. The indicated BLCA cells were injected subcutaneously into nude mice. **C** H and E staining of primary tumour borders. Scale bar 300 μm. **D** Representative images and quantification of microvessel density (MVD) in tumours indicated by CD31 staining. Scale bar 300 μm. **E** IVIS detection of lung metastases in nude mice (left) and their quantitation (right). **F** Bright-field images and quantification of lung metastases. **G** H and E of lung metastases. Scale bar 500 μm. A two-tailed *t*-test was used for statistical analysis. Error bars represent the mean ± SD of three independent experiments. **P < 0.01

**Fig. 3:**
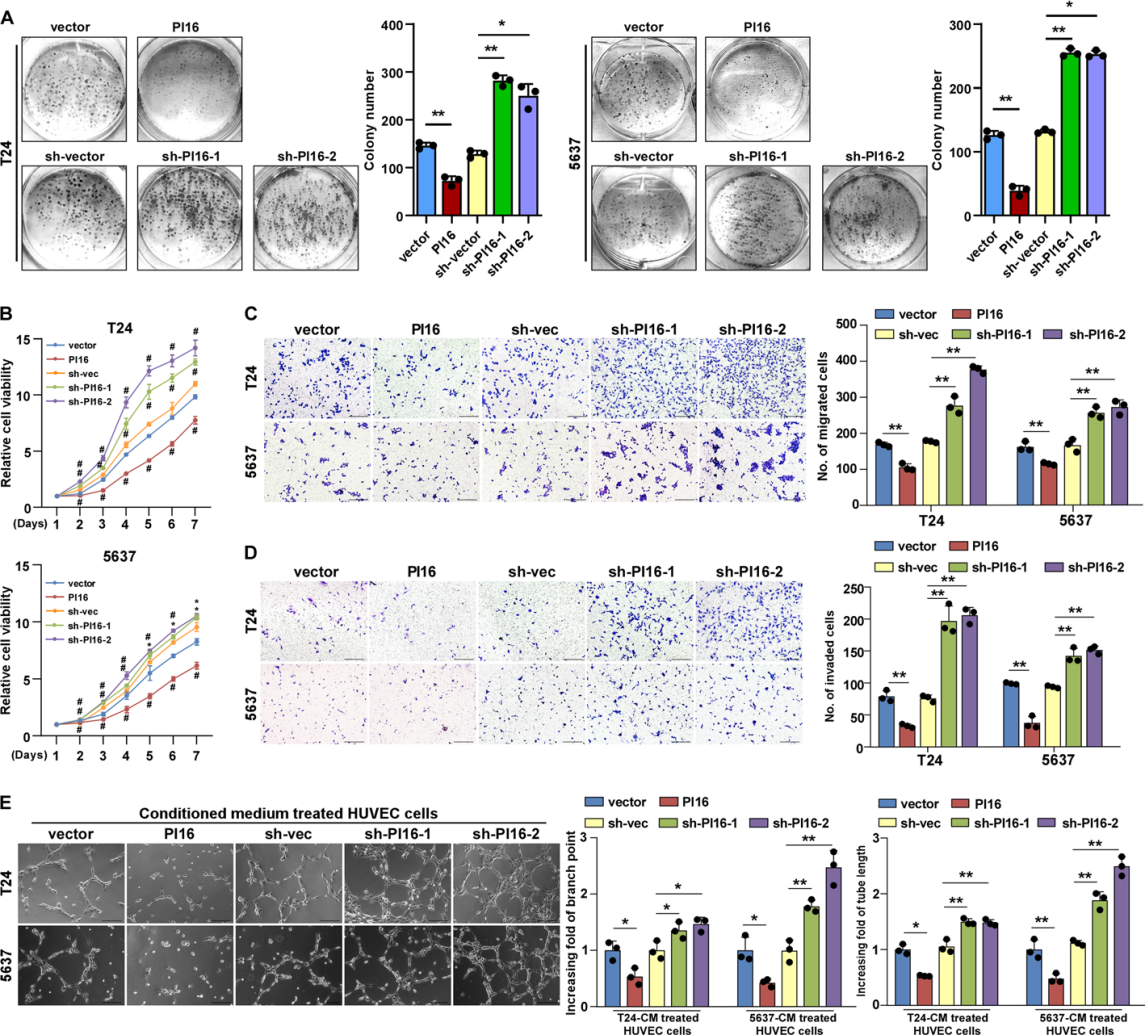
PI16 suppresses BLCA metastasis in vitro. **A** Representative bright-field microscopic images (left) and quantification (right) of colony formation for indicated cells. **B** MTS assayed PI16’s effect on indicated cell viability. ^#^*P* < 0.01. **C**, **D** Representative images (left) and quantification (right) of migrated (**C**) and invaded (**D**) cells were analysed in a transwell assay. Scale bar 100 μm. **E** Representative images and quantification of tubules formed after HUVECs were cultured on matrigel-coated plates with BLCA cell conditioned medium (CM). Scale bar 100 μm. A two-tailed *t*-test was used for statistical analysis. Error bars represent the mean ± SD of three independent experiments. *P < 0.05; **P < 0.01

GSEA analysis showed that PI16 expression negatively correlated with metastasis-related genes (Additional file 1: Fig. S2A). To confirm PI16’s biological role, we used an in vivo murine model (MB49 cells; Additional file 1: Fig. S2, B, C). IVIS imaging showed PI16 overexpression inhibited tumour growth, while PI16 knockdown promoted tumour growth (Fig. 2A, Additional file 1: Fig. S2D). PI16-overexpressing tumours grew slower and were smaller/lighter. PI16-knockdown tumours were larger/heavier (Fig. 2B, Additional file 1: Fig. S2E). Haematoxylin and eosin (H and E) staining showed PI16-overexpressing tumours had well-defined borders, indicating low invasiveness. PI16-knockdown tumours showed spine-like structures penetrating muscle, indicating high invasiveness (Fig. 2C). CD31 staining showed lower microvessel density (MVD) in PI16-overexpressing tumours, indicating PI16 inhibits angiogenesis (Fig. 2D). In a metastasis model, mice injected with PI16-overexpressing cells had few lung metastases. Mice injected with PI16-knockdown cells had significantly more metastases (Fig. 2E–G, Additional file 1: Fig. S2F), suggesting PI16 suppresses BLCA invasion and metastasis in vivo. In vitro, we established T24 and 5637 cells with stable PI16 overexpression or knockdown (Additional file 1: Fig. S3A). PI16 overexpression inhibited and PI16 knockdown promoted cell viability and growth (Fig. 3A, B). Wound healing and transwell assays showed PI16 overexpression reduced and PI16 knockdown increased motility (Additional file 1: Fig. S3B, Fig. 3C). Transwell matrix penetration showed PI16 overexpression decreased and PI16 knockdown increased invasiveness (Fig. 3D). Angiogenesis assays showed PI16 overexpression inhibited and PI16 knockdown promoted tube formation and HUVEC migration (Fig. 3E; Additional file 1: Fig. S3C), suggesting that PI16 inhibits BLCA migration and invasion in vitro. In summary, our results demonstrate that PI16 inhibits tumorigenicity, angiogenesis, invasion and metastasis of BLCA cells in vivo and in vitro. PI16 may act as a tumour suppressor in BLCA.

### PI16 inhibits NF-κB activity in BLCA

**Fig. 4:**
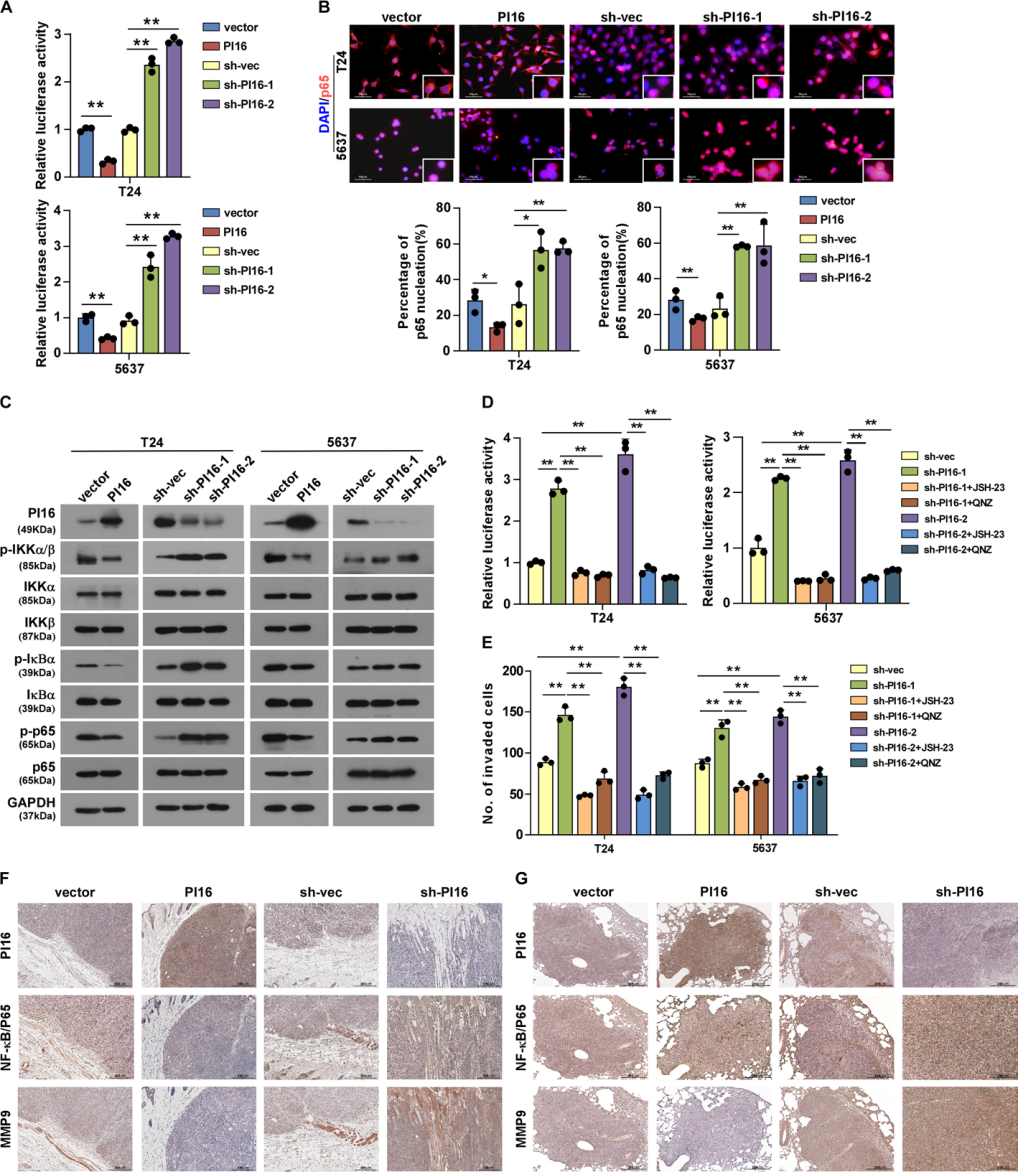
PI16 inhibits NF-κB activity. **A** NF-κB luciferase reporter activity in indicated cells. **B** Immunofluorescence of NF-κB/p65 subcellular localization in indicated cells (top); quantification of NF-κB/p65 nuclear localization (bottom). Scale bar 50 μm. **C** Western blot of NF-κB pathway regulators in indicated cells. **D** NF-κB luciferase reporter activity in indicated cells with NF-κB inhibitor. **E** Quantification of the indicated invaded cells was analysed in a transwell matrix penetration assay. JSH-23 (20 μM, 48 h) and QNZ (1 μM, 48 h) were used. **F** IHC of subcutaneous tumours (PI16, NF-κB/p65, MMP9). Scale bar 300 μm. **G** IHC of metastatic lung tumour nodes (PI16, NF-κB/p65, MMP9). T24, 5637: in vitro; MB49: in vivo model. Scale bar 200 μm. A two-tailed *t*-test was used for statistical analysis. Error bars represent the mean ± SD of three independent experiments. *P < 0.05, **P < 0.01

GSEA showed PI16 expression negatively correlates with NF-κB activation (Additional file 1: Fig. S4A). Luciferase reporter assays showed PI16 overexpression inhibited and PI16 knockdown promoted NF-κB transcriptional activity (Fig. 4A). PI16 overexpression inhibited and PI16 knockdown promoted NF-κB/p65 nuclear translocation (Fig. 4B; Additional file 1: Fig. S4B). Accordingly, NF-κB-regulated gene expression decreased with PI16 overexpression and increased with PI16 knockdown (Additional file 1: Fig. S4C). Western blot showed PI16 overexpression decreased and PI16 knockdown increased phosphorylation of IKKα/β, IκBα and p65, with no change in total protein (Fig. 4C). To confirm NF-κB’s role, we used NF-κB inhibitors in vitro. Inhibitors blocked NF-κB activation (Fig. 4D) and motility and invasion (Additional file 1: Fig. S4, D–F; Fig. 4E) induced by PI16 knockdown. IHC of subcutaneous tumours showed NF-κB/p65 nuclear expression and MMP9 (a marker of invasion and NF-κB target) decreased in the PI16 overexpression group but increased in the PI16 knockdown group (Fig. 4F). Similarly, in clinical metastatic lung tumours, NF-κB/p65 nuclear expression and MMP9 decreased in the PI16 overexpression group but increased in the PI16 knockdown group (Fig. 4G). In summary, our results show PI16 overexpression inhibits and PI16 knockdown activates NF-κB signalling. NF-κB mediates the effects of PI16 on BLCA motility, invasion and metastasis.

### PI16 suppresses NF-κB signalling through ANXA1-dependent ubiquitination of NEMO

**Fig. 5:**
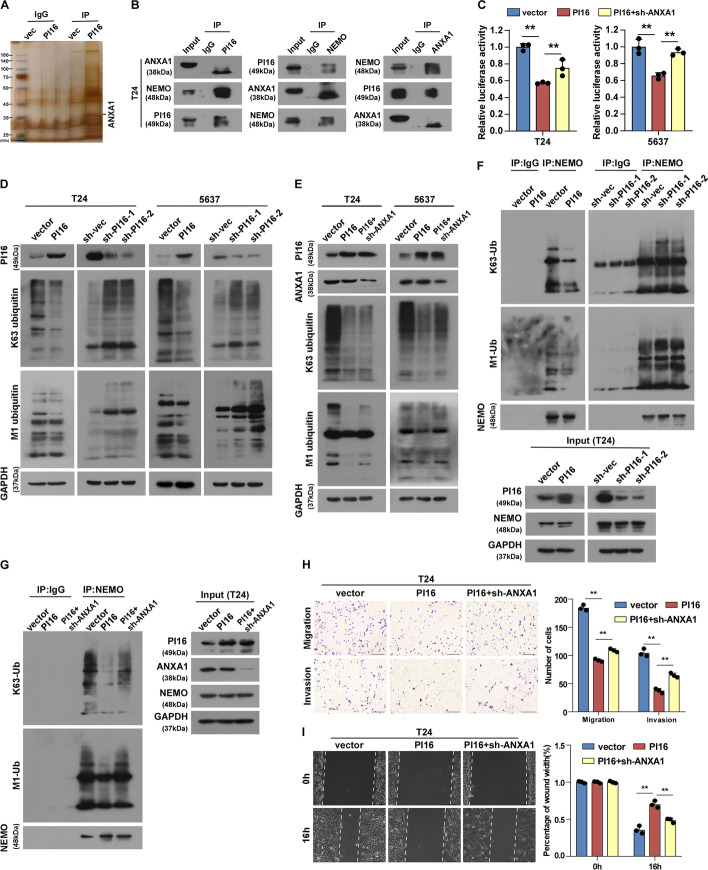
PI16 inhibits NF-κB activity by blocking ANXA1-dependent NEMO ubiquitination. **A** PI16-transfected T24 lysates immunoprecipitated with anti-PI16; silver stain showed PI16/ANXA1 interaction. **B** PI16-transfected T24 lysates immunoprecipitated with anti-PI16, anti-ANXA1 and anti-NEMO; co-IP showed PI16/ANXA1/NEMO interaction. **C** NF-κB luciferase reporter activity in indicated cells. **D**, **E** Western blot of K63-Ub and M1-Ub in indicated cells. **F**, **G** Anti-NEMO immunoprecipitated T24 lysates; immunoblot showed K63-Ub and M1-Ub. **H** Transwell migration (upper) and invasion (lower) assay images and quantitation of T24 cells. Scale bar 100 μm. **I** Wound healing assay images (left) and quantitation (right) of T24 cells. Scale bar 100 μm. A two-tailed *t*-test was used for statistical analysis. Error bars represent the mean ± SD of three independent experiments. **P < 0.01

Co-immunoprecipitation (co-IP) and mass spectrometry analyses identified ANXA1 as a protein that interacts with PI16 (Fig. 5A, B; Additional file 1: Fig. S5, A, B). We validated this interaction using exogenous co-IP and immunofluorescence co-localization assays (Additional file 1: Fig. S5, C, D). ANXA1 knockdown attenuated PI16-mediated NF-κB inhibition (Fig. 5C), although ANXA1 and NEMO protein levels did not change with PI16 expression (Additional file 1: Fig. S5E). It has been reported that silencing ANXA1 prevents the interaction between NEMO and RIP1, which in turn inhibits NF-κB activation [16]. Our result showed PI16 interacted with NEMO (Fig. 5B; Additional file 1: Fig. S5B). NEMO recognizes K63 and M1-linked polyubiquitination, which are critical for activating NF-κB signalling [17, 18]. K63-Ub and M1-Ub decreased with PI16 overexpression, but increased with PI16 knockdown (Fig. 5D). ANXA1 knockdown rescued this decrease (Fig. 5E). Co-IPs showed PI16 overexpression reduced NEMO K63-Ub and M1-Ub; ANXA1 knockdown replenished NEMO K63-Ub and M1-Ub (Fig. 5F, G; Additional file 1: Fig. S5, F, G). ANXA1 knockdown attenuated the reduced motility and invasion from PI16 overexpression (Fig. 5H, I; Additional file 1: Fig. S5, H-I). Together, these results suggest PI16 suppresses NF-κB signalling through ANXA1-dependent multiple-site ubiquitination of NEMO.

### PI16 inhibits NEMO ubiquitination and NF-κB activation by suppressing LUBAC expression

**Fig. 6:**
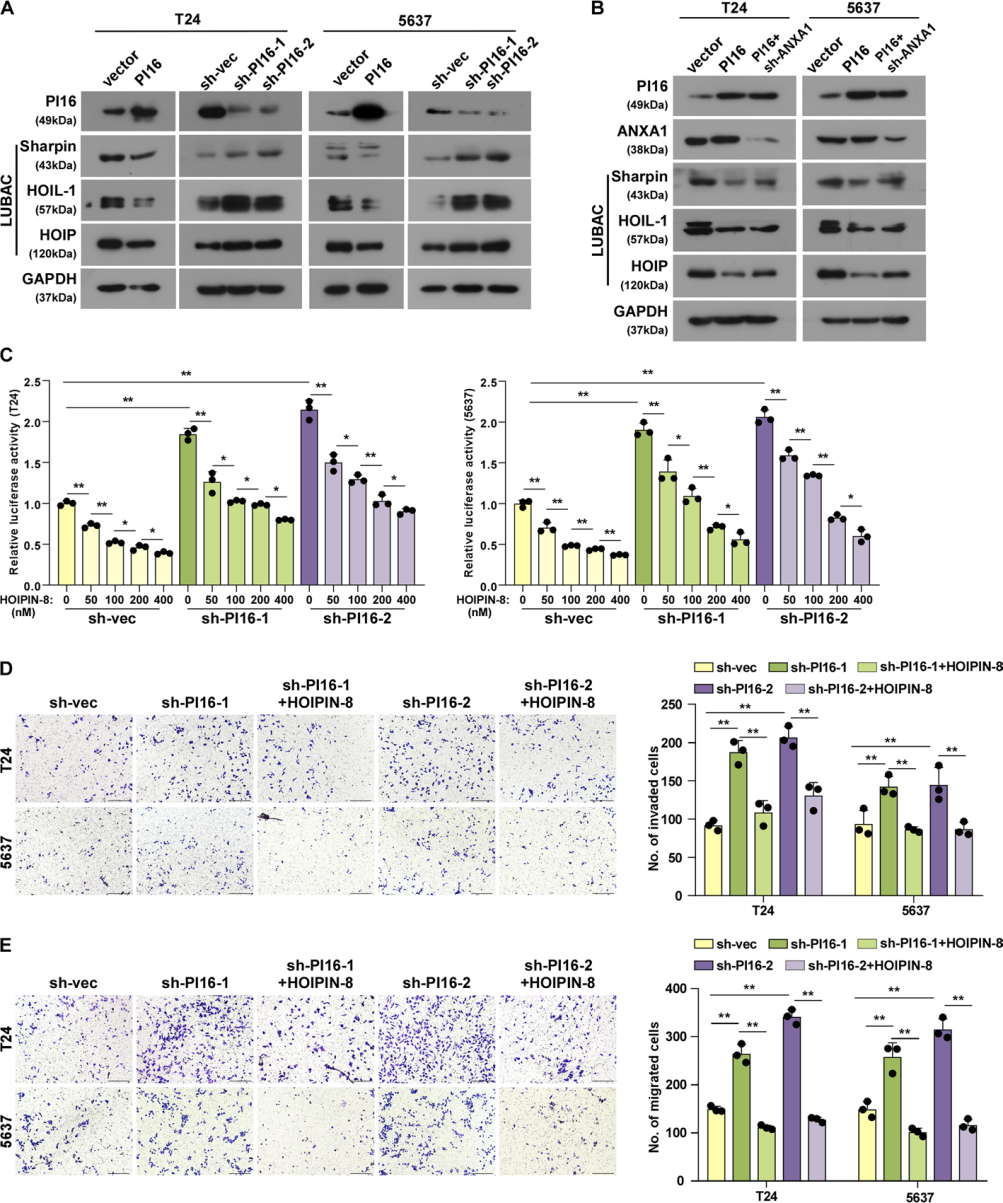
PI16 inhibits NF-κB activity by blocking LUBAC expression through ANXA1 binding. **A** Western blot of LUBAC components in indicated cells. **B** Western blot of ANXA1 and LUBAC components in indicated cells. **C** NF-κB luciferase reporter activity in indicated cells with HOIPIN-8 (0 nM, 50 nM,100 nM, 200 nM and 400 nM, for 48 h). **D**, **E** Transwell invasion (**D**) and migration (**E**) assay images and quantitation for indicated cells with HOIPIN-8 (400 nM). Scale bar 100 μm. A two-tailed *t*-test was used for statistical analysis. Error bars represent the mean ± SD of three independent experiments. *P < 0.05, **P < 0.01

**Fig. 7:**
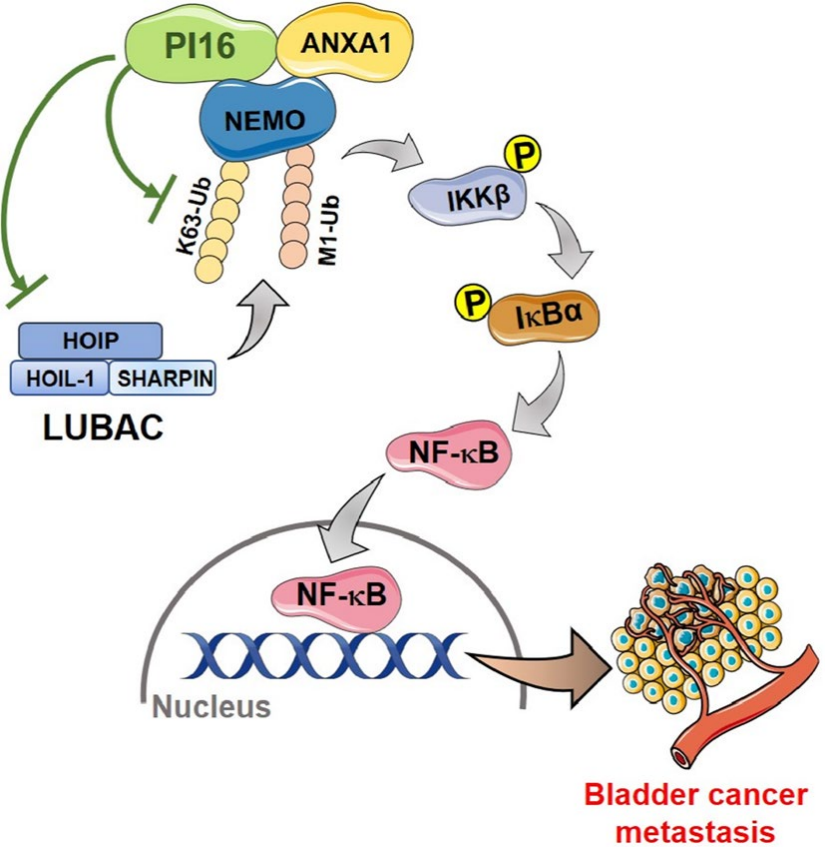
Proposed model diagram of PI16 inhibition of NF-κB signalling pathway and BLCA metastasis

Previously, K63-linked ubiquitination by tumour necrosis factor (TNF) receptor-associated factor 6 (TRAF6) recruited the linear ubiquitin assembly complex (LUBAC) consisting of SHANK-associated RH domain interactor (Sharpin), Ring finger Protein 31 (HOIP) and RANBP-type and C3HC4-type zinc finger containing 1 (HOIL-1), which generates M1-linked ubiquitination. This binds NEMO, inducing IKK complex changes and activating NF-κB signalling activation [19, 20]. We found HOIP, HOIL-1 and Sharpin decreased with PI16 overexpression but increased with PI16 knockdown (Fig. 6A). ANXA1 knockdown rescued this decrease (Fig. 6B). The LUBAC inhibitor HOIPIN-8 decreased NF-κB activation from PI16 knockdown in a dose-dependent manner (Fig. 6C). HOIPIN-8 also inhibited motility and invasion induced by PI16 knockdown (Fig. 6, D–E). This indicates LUBAC mediates how PI16 suppresses NF-κB and motility and invasion. In conclusion, PI16 acts as a BLCA suppressor by inhibiting NF-κB through ANXA1-dependent suppression of NEMO K63- and M1-ubiquitination, ultimately inhibiting invasion and metastasis (Fig. 7).

## Discussion

Activation of proto-oncogenes and inactivation of tumour suppressors are considered to be key events in tumour initiation and development. Previous studies reported that loss of PI16 expression was associated with the recurrence of early prostate cancer [7, 21], while Ma et al. [22] found that PI16 was overexpressed in ovarian cancer cells. These findings supported the notion that the oncogenic and anti-tumour role of PI16 in the tumour field is dependent on tumour type. Currently, we identified that the downregulation of PI16 was positively correlated with unfavourable survival and prognosis of patients with BLCA and could serve as a tumour suppressor in BLCA progression. Notably, it has been reported that PI16 was one of the most abundant proteins secreted by the epithelial cells of the prostate lumen and was also present in other body fluids, and the serum level of PI16 can predict the risk level of prostate cancer [23, 24], further elucidating the fundamental potential of PI16 as a minimally invasive diagnostic marker. The potential diagnostic value of PI16 in the body fluids of patients with BLCA is worth further investigation.

The NF-κB is an important transcriptional regulator in the process of intracellular signal transduction. NF-κB can be activated by a variety of ligand/receptor complexes, including the TNF receptor superfamily, IL-1 receptor/Toll-like receptor superfamily, LPS and similar stimuli [25, 26]. Meanwhile, it is also inhibited by many negative regulators [27, 28]. PTEN, a tumour suppressor, was downregulated in lung cancer and pancreatic ductal adenocarcinoma, enhancing the cancer invasiveness by NF-κB pathway transduction [29, 30]. Overexpression of LRRC26 in breast cancer inhibits NF-κB activity and suppresses tumorigenesis and lung metastasis [31]. Our study confirmed that PI16 exerted its inhibitory role in the malignant progression and metastasis of BLCA by inhibiting NF-κB activation, which may provide new clues for the understanding of tumours associated with abnormal NF-κB activation, and targeting PI16 may achieve control of abnormal NF-κB activation in tumours.

Our study found that PI16 inhibited NF-κB activation by binding to ANXA1, which is a member of the annexin superfamily and initially identified as a glucocorticoid-regulated anti-inflammatory protein relevant to the innate and adaptive immune responses [32, 33]. Current studies on ANXA1 in BLCA were divergent. According to the study of Cui et al., ANXA1 was downregulated in murine-derived bladder cell carcinoma [34]. However, some studies demonstrated that ANXA1 was upregulated in BLCA and promoted BLCA progression [35, 36]. In our study, we found that silencing ANXA1 blocked the inhibitory effect of PI16 on NF-κB activity and cell invasion, whereas the expression level of ANXA1 remained unchanged in the BLCA cells with changed expression of PI16, indicating that ANXA1 might perform as an imperative chaperon of PI16 in the regulation of NF-κB signalling pathway. Therefore, further determining ANXA1’s role could significantly impact understanding PI16 and BLCA.

The regulation of ubiquitination modifications is closely related to the activation of the NF-κB signalling pathway. Recently, the role of M1-Ub modifications generated by the LUBAC complex has been representing a more important function in the regulation of the NF-κB pathway [37, 38]. It has been reported that the inhibition of LUBAC complex dysfunction could be considered an effective treatment for tumours [39]. Ruiz et al. found that inhibition of LUBAC effectively inhibits NF-κB activation to re-sensitize squamous cell lung cancer to cisplatin [40]. LUBAC accelerates B-cell lymphoma development and inhibition of LUBAC inhibits the progression of B-cell lymphoma with NF-κB activation [41]. LUBAC inhibitor HOIPIN-8 inhibits breast cancer cell proliferation and clone formation by blocking EGFR-mediated NF-κB activation [42]. Although there is currently no LUBAC inhibitor available for effective in vivo use, we have demonstrated in our in vitro experiments that the LUBAC inhibitor HOIPIN-8 can significantly suppress NF-κB activation induced by PI16 and the motility and invasion of BLCA cells. Furthermore, our study suggests that PI16 may act as a potential LUBAC inhibitor to exert its tumour suppressor function, and the role of PI16 in in vivo models also suggests the promising potential of targeting the LUBAC pathway for anti-tumour therapy. Together, these findings provide the rationale for developing PI16 as a potential therapeutic strategy against BLCA.

## Conclusions

In our study, we discovered that PI16 plays a vital role in suppressing the development of tumours and the progression of metastasis in BLCA by inhibiting the activity of NF-κB through ANXA1-dependent ubiquitination of NEMO. These findings provide valuable insights into the molecular mechanisms underlying metastatic BLCA and suggest that PI16 could be a promising target for diagnosis and treatment. Further research into the role of PI16 in BLCA could lead to the development of novel therapeutic strategies.

## Supplementary Information

**Additional file 1. **Supplementary Materials and Methods, Tables and Figures.

## Data Availability

For the survival analysis of patients, the TCGA datasets were used. All other data supporting the findings of this study are available within the article and its supplementary information files and on reasonable request from the corresponding author.

## Abbreviations

BLCA: Bladder cancer
PI16: Peptidase inhibitor 16
NF-κB: Nuclear factor κB
LUBAC: Linear ubiquitin chain assembly complex
ANXA1: Annexin A1
mRNA: Messenger RNA
IgG: Immunoglobulin G
MOD: Mean optical density
MVD: Microvessel Density
DMSO: Dimethyl sulfoxide
DTT: Dithiothreitol
TEMED: *N′N′N′N′*-Tetramethylethylenediamine
SDS: Sodium dodecyl sulfate
EDTA: Ethylenediaminetetraacetic acid
FBS: Fetal bovine serum
PBS: Phosphate-buffered saline
DMEM: Dulbecco’s Modified Eagle Medium
GSEA: Gene set enrichment analysis
TCGA: The Cancer Genome Atlas
qPCR: Quantitative real-time PCR
IHC: Immunohistochemistry
SPSS: Statistical package for the social science
K63-Ub: K63 ubiquitination
M1-Ub: M1 ubiquitination
