# Supplementary material for: Peptidase inhibitor (PI16) impairs bladder cancer metastasis by inhibiting NF-κB activation via disrupting multiple-site ubiquitination of NEMO
Source: Cell Mol Biol Lett. 2023 Jul 31;28:62. doi: 10.1186/s11658-023-00465-6 (PMC10388466; doi:10.1186/s11658-023-00465-6)

Supplementary Materials and Methods

Colony formation assay

Cells were plated in 6-well plates at a density of 1000 cells/well. After 10-14 days of culture, cells were fixed with 4% paraformaldehyde and stained with 0.5% crystal violet. Visible colonies containing more than 50 cells were counted. Quantification of colony numbers normalized to the control group. All experiments were performed in triplicate.

Wound healing assay

Cells were first digested and counted and seeded on a 24-well plate, cell sink contract at 90% density, and then starve cells with basal culture medium for 4 hours. The cell monolayer was then scraped with the tip of a 10 μL sterile pipette to form a straight wound, floating cells were washed with phosphate-buffered saline (PBS) and the cells were cultured in a medium containing 10% FBS. The images were then captured using an inverted microscope and the migration process was recorded.

Immunofluorescence staining assay

After the cell crawls were soaked in 75% alcohol and placed on an ultraviolet light table for 30 minutes, the BLCA cells were grown on coverslips of 24-well plates. After standing for 24 hours, the culture medium was discarded. After washing twice with PBS, cells were fixed with 4% paraformaldehyde for 15 minutes and treated with 0.1% TritionX-100 for 5 minutes, then blocked with 10% BSA for 30 minutes. The primary antibody p65 (#8242), HA (#3724) and Flag (#8146) (Cell Signaling) was incubated for 1 hour at room temperature. After washing with PBS, the coverslips were incubated with anti-rabbit secondary antibodies coupled with fluorescent dyes (Cell Signaling, #4413, #4408) for 1 hour. The nuclei were counterstained with 2-(4-amidinophenyl)-6-indolecarbamidine (DAPI) (Thermo, #62248) for 10 minutes. After washing twice with PBS, the films were sealed after the r dropwise addition of anti-fading reagent (Thermo, #36974) and kept in the dark for observation under the microscope and photo-taking of the soil.

Co-immunoprecipitation and mass spectrometry analysis

For immunoprecipitation, dynabeads (Millipore, #LSKMAGAG10) coupled with PI16-antibody (1 μg antibody/100μg protein) were prepared and mixed with cell lysates harvested from T24 cells transfected with PI16 plasmids. After rotating for 1 hour at room temperature, the immune complexes separated from dynabeads were washed with lysis buffer and then suspended in SDS blue loading buffer. Lysis was performed at 70°C for 10 minutes and the protein was assayed by Western blotting. The Fast Silver Stain Kit (Beyotime, Shanghai, # P0017S) was used for silver staining according to the manufacturer's instructions. Mass spectrometry analysis was further performed by Wininovate Bio Company (Shenzhen). The detailed procedure is as follows. Aliquots of proteins were mixed with 200 μL of 8 M urea in Nanosep Centrifugal Devices (PALL). The device was centrifuged at 14,000 g at 20℃ for 20 minutes. All following centrifugation steps were performed applying the same conditions allowing maximal concentration. The concentrate was diluted with 200 μL of 8 M urea in 0.1 M Tris-HCl, pH 8.5, and the device was centrifuged. Subsequently, 100 μL of 0.05 M iodoacetamide in 8 M urea in 0.1 M Tris-HCl, pH 8.5 were added to the concentrate followed by centrifugation. The resulting concentrate was diluted with 200 μL 8 M urea in 0.1 M Tris-HCl, pH 8.0, and concentrated again. This step was repeated 2 times, and the concentrate was subjected to proteolytic digestion. The digests were collected by centrifugation, and the filter device was rinsed with 50 ml 0.5 M NaCl and centrifuged. The lyophilized peptide fractions were re-suspended in 2% acetonitrile containing 0.1% formic acid, and 2 μL aliquots of which were loaded into a nano Viper C18 (3 μm, 100 Å) trap column. The online Chromatography separation was performed on the Easy-nLC 1000 system (ThermoFisher). The trapping and desalting procedure was carried out at a volume of 3 μL for 5 minutes with 100% solvent A (water/acetonitrile/formic acid (98/2/0.1%; B, 2/98/0.1%)). Then, an elution gradient of 8-38% solvent B in 60 minutes was used on an analytical column (50 μm ×15 cm C18-3 μm 100 Å). IDA (information-dependent acquisition) mass spectrum techniques were used to acquire tandem MS data on a ThermoFisher Q exactive mass spectrometer (ThermoFisher, USA) fitted with a Nano Flex ion source. Data were acquired using an ion spray voltage of 1.9 kV, and an interface heater temperature of 275℃. The MS was operated with FULL-MS scans. For IDA, survey scans were

acquired in 250 ms and up to 20 product ion scans (50 ms) were collected. Only spectra with a charge state of 2-4 were selected for fragmentation by higher-energy collision energy. Dynamic exclusion was set for 25s. The MS/MS data were analyzed for protein identification and quantification using PEAKS. The local false discovery rate was 1.0% after searching against the Homo sapiens sequence with a maximum of two missed cleavages and one missed termini cleavage. The following settings were selected: oxidation (M), Acetylation (Protein N-term), Deamidation (NQ), Oxidation (M), Pyro-glu from E, Pyro-glu from Q for variable modifications as well as fixed carbamidomethylating of cysteine. Precursor and fragment tolerance were set to 10 ppm and 0.05 Da, respectively.

Nuclear protein extraction assay

After the cells were grown to a certain density, a nucleoplasm protein extraction kit (Thermo, #36974) was used to extract nuclear proteins and cytosol proteins. After washing the cells twice with PBS, collect the cell suspension into a centrifuge tube after trypsin digestion, centrifuge at 800 rpm for 5 minutes at room temperature, gently resuspend with 100 μL 1× cytoplasmic extraction reagent I, vortex rapidly for 15 seconds, and incubate for 15 minutes on ice. Add 5.5 μL of pre-chilled Cytoplasmic Extraction Reagent II, vortex quickly for 15 seconds, and incubate on ice for 1 minute. The lysate was centrifuged at 16,000 g for 10 minutes at 4°C and the supernatant (cytoplasmic extract) was immediately transferred to a clean EP tube. Nuclear proteins were then extracted with 50 μL of pre-chilled nuclear protein extraction reagent. The homogenate was vortexed rapidly for 10 seconds, placed on ice for 10 minutes, vortexed for 15 seconds every 10 minutes for a total of 40 minutes, and then centrifuged at 16000g for 10 minutes at 4°C. Immediately transfer the supernatant to EP tubes for BCA quantification and store at -80°C until use. Primary antibody p65 (Cell Signaling, #8242) was used to detect nuclear and cytoplasmic p65 expression. p84 (Abcam, #ab125019) was used as a nuclear loading control and β-actin (Cell Signaling, #3700) was used as a cytoplasmic loading control.

Supplementary figure legends

Supplementary Figure 1. (A) TNMplot database (*http://www.tnmplot.com*) shows the expression of PI16 in different tissues. (B) Representative images of IHC analysis of PI16 expression. Scale bar, 500 μm. (C) Quantitative PCR and western blot analysis of PI16 expression in eight human BLCA cell lines and a normal immortalized bladder epithelial cell line SV-HUC-1. A two-tailed t-test was used for statistical analysis. Error bars represent the mean ± SD of three independent experiments. *, P <0.05; ** P <0.01.

Supplementary Figure 2. PI16 suppresses metastasis of BLCA. (A) The correlation of PI16 expression with gene signatures of metastasis through GSEA analysis. (B) Western blot analysis of PI16 expression to confirm the efficacy of PI16-shRNA. shRNA#4 was chosen for the *in vivo* experiments shown as sh-PI16, and shRNA #1 and #4 were chosen for the *in vitro* experiments, shown as sh-PI16-1 and sh-PI16-2 based on the shRNA efficacy. (C) Western blot analysis of the murine BLCA cell line MB49 stably transduced with PI16-vector (vec), PI16, shRNA-vector (sh-vec), or shRNA-PI16 (sh-PI16). (D) Representative images of *in vivo* imaging system (IVIS) detection of subcutaneous tumours in nude mice. (E) Representative images of subcutaneous tumours, the volume and weight of tumours. The indicated BLCA cells were injected subcutaneously into nude mice. (F) IVIS detection of lung metastases in nude mice (left) and quantitation (right). A two-tailed t-test was used for statistical analysis. Error bars represent the mean ± SD of three independent experiments. **, P <0.01.

Supplementary Figure 3. PI16 suppresses metastasis of BLCA *in vitro*. (A) Western blot analysis of BLCA cells stably transduced with PI16-vector (vec), PI16, shRNA-vector (sh-vec), shRNA-PI16-1 (Ri#1), or shRNA-PI16-2 (Ri#4). (B) Representative images (left) and quantification (right) of wound healing assay of the indicated cells. Scale bar, 100 μm. (C) Representative images (left) and quantification (right) of migrated HUVECs analyzed in a transwell assay. A two-tailed t-test was used

for statistical analysis. Scale bar, 100 μm. Error bars represent the mean ± SD of three independent experiments. **P < 0.01.

Supplementary Figure 4. PI16 inhibits NF-κB pathway activity. (A) The correlation of PI16 expression with gene signatures of NF-κB pathway through GSEA analysis. (B) Western blot analysis of the subcellular localization of NF-κB in indicated cells. p84 was used as a nuclear loading control. β-actin was used as a cytoplasmic loading control. (C) Quantitative PCR analysis of NF-κB regulated genes in the indicated cells. (D) Representative images of invaded cells were analyzed in a transwell assay. Scale bar, 100 μm. (E) Representative images (left) and quantification (right) of wound healing assay of the indicated cells. Scale bar, 100 μm. (F) Representative images (left) and quantification (right) of migrated cells were analyzed in transwell assay, cells were treated with JSH-23 and QNZ. Scale bar, 100 μm. A two-tailed t-test was used for statistical analysis. Error bars represent the mean ± SD of three independent experiments. ** P < 0.01.

Supplementary Figure 5. PI16 restrains the NF-κB pathway through ANXA1-dependent NEMO ubiquitination. (A) Lysates from T24 cells transfected with PI16 were immunoprecipitated with an anti-PI16 antibody, the mass spectrometry peptide sequencing revealed an interaction between SBSN and ANXA1. (B) 5637 cells transfected with PI16 plasmid, and antibodies against PI16, ANXA1 and NEMO were used to perform co-IP. (C) PI16-Flag and ANXA1-His transfected 293FT lysates immunoprecipitated with anti-Flag and anti-His; co-IP showed PI16/ANXA1 interaction. (D) Immunofluorescence co-localization assays performed on PI16-Flag and ANXA1-His transfected 293FT cells showed PI16/ANXA1 co-localization. (E) Western blot analysis of the effect of PI16 on ANXA1 and NEMO expression in the indicated cells. (F-G) 5637 cell lysates were immunoprecipitated with an anti-NEMO antibody, followed by immunoblotting with an anti-K63 or anti-M1 ubiquitin antibody. (H) Representative images (left) and quantification (right) of 5637 migrated cells analyzed in a transwell assay and invaded cells analyzed in a transwell matrix penetration assay. Scale bar, 100 μm. (I) Representative images (left) and

quantification (right) of wound healing assay of 5637 cells. Scale bar, 100 μm. A two-tailed t-test was used for statistical analysis. Error bars represent the mean ± SD of three independent experiments. $**P < 0.01$.

Supplementary Table 1. Clinicopathologic characteristics of bladder cancer

Factor	No.	(%)
**Gender**		
Male	58	85.3
Female	10	14.7
**Age (years)**		
≤68	35	51.5
>68	33	48.5
**Clinical stage**		
I	6	8.8
II	13	19.1
III	24	35.3
IV	11	16.2
Information missing	14	20.6
**T classification**		
T1	12	17.6
T2	16	23.5
T3	25	36.8
T4	5	7.4
Information missing	10	14.7
**Lymph node invasion**		
Yes	11	16.2
No	47	69.1
Information missing	10	14.7
**Vital status**		
Alive	36	52.9
Dead	32	47.1

**Expression of PI16 （MOD）**		
Low expression	34	50.0
High expression	34	50.0

Supplementary Table 2. Cox-regression analysis for univariate and multivariate analysis of different prognostic parameters in bladder patients (determined by MOD value)

	Univariate analysis			Multivariate analysis		
	P	Relative risk [Exp(B)]	95% confidence interval	*P*	Relative risk [Exp(B)]	95% confidence interval
**Gender**	0.440	0.704	0.288-1.729			
**Age**	0.797	1.005	0.968-1.044			
**Clinical stage**	0.029	1.651	1.053-2.589			
**T classification**	0.142	1.370	0.900-2.085			
**N classification**	0.043	2.516	1.031-6.140			
**Expression of PI16(MOD)**	0.013	0.398	0.193-0.821	0.035	0.228	0.058-0.904

Supplementary Table 3. Sequences of primers and si/sh-RNA

PI16 Forward sequence	CTGGTGTGCAACTATGAGCCTC
PI16 Reverse sequence	GGCAAATCCTGAGCATCTTCCG
IL-6 Forward sequence	AGACAGCCACTCACCTCTTCAG
IL-6 Reverse sequence	TTCTGCCAGTGCCTCTTTGCTG
IL-8 Forward sequence	GAGAGTGATTGAGAGTGGACCAC
IL-8 Reverse sequence	CACAACCCTCTGCACCCAGTTT
BCL-XL Forward sequence	GCCACTTACCTGAATGACCACC
BCL-XL Reverse sequence	AACCAGCGGTTGAAGCGTTCCT
CCND Forward sequence	TCTACACCGACAACTCCATCCG
CCND Reverse sequence	TCTGGCATTTTGGAGAGGAAGTG
VEGFC Forward sequence	GCCAATCACACTTCCTGCCGAT
VEGFC Reverse sequence	GGTCTTGTTCGCTGCCTGACA
MMP9 Forward sequence	GCCACTACTGTGCCTTTGAGTC
MMP9 Reverse sequence	CCCTCAGAGAATCGCCAGTACT
TNFα Forward sequence	CTCTTCTGCCTGCTGCACTTTG
TNFα Reverse sequence	ATGGGCTACAGGCTTGTCACTC
MYC Forward sequence	CCTGGTGCTCCATGAGGAGAC
MYC Reverse sequence	CAGACTCTGACCTTTTGCCAGG
CCL20 Forward sequence	AAGTTGTCTGTGTGCGCAAATCC
CCL20 Reverse sequence	CCATTCCAGAAAAGCCACAGTTTT
FGF1 Forward sequence	ATGGCACAGTGGATGGGACAAG
FGF1 Reverse sequence	TAAAAGCCCGTCGGTGTCCATG
CXCL1 Forward sequence	AGCTTGCCTCAATCCTGCATCC
CXCL1 Reverse sequence	TCCTTCAGGAACAGCCACCAGT
GAPDH Forward sequence	GTCTCCTCTGACTTCAACAGCG
GAPDH Reverse sequence	ACCACCCTGTTGCTGTAGCCAA
ANXA1-siRNA	GCGTCAACAGATCAAAGCA

PI16-shRNA-Ri1	CCGGGAGGAGACCAACATCGAATTACTCGAG<br>TAATTCGATGTTGGTCTCCTCTTTTTTG
PI16-shRNA-Ri2	CCGGGCAGACAAAGTGACAGACAAACTCGA<br>GTTTGTCTGTCACTTTGTCTGCTTTTTTG

Supplementary Figure 1

A

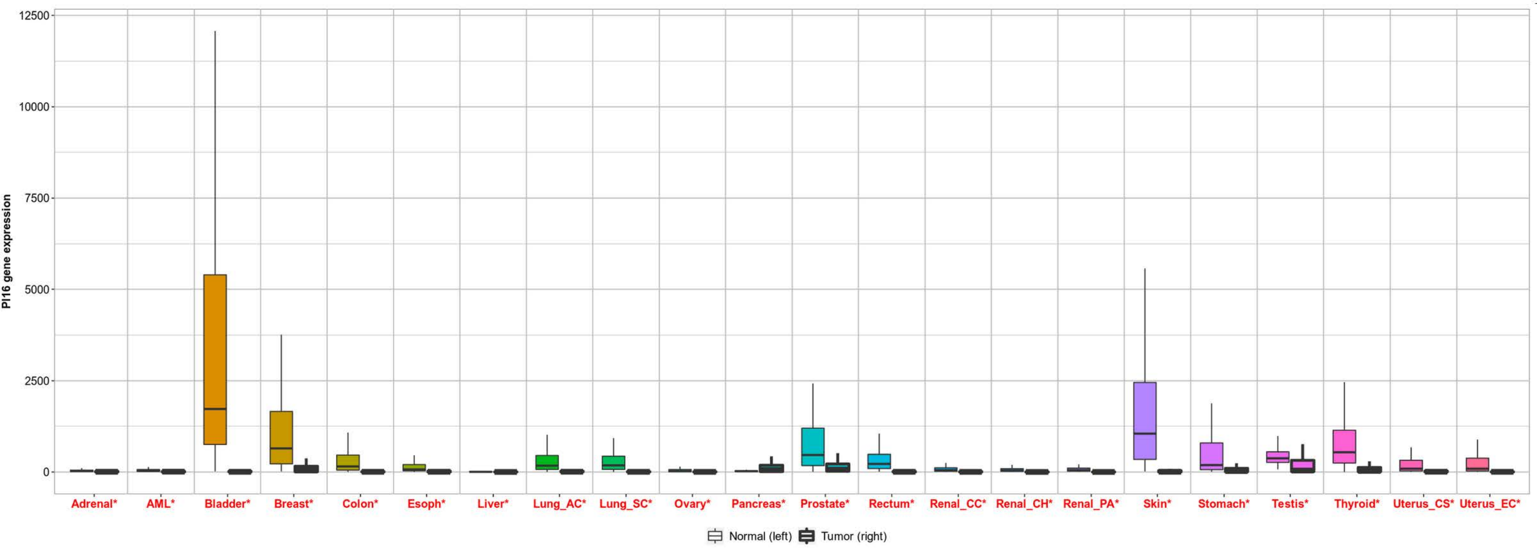

B

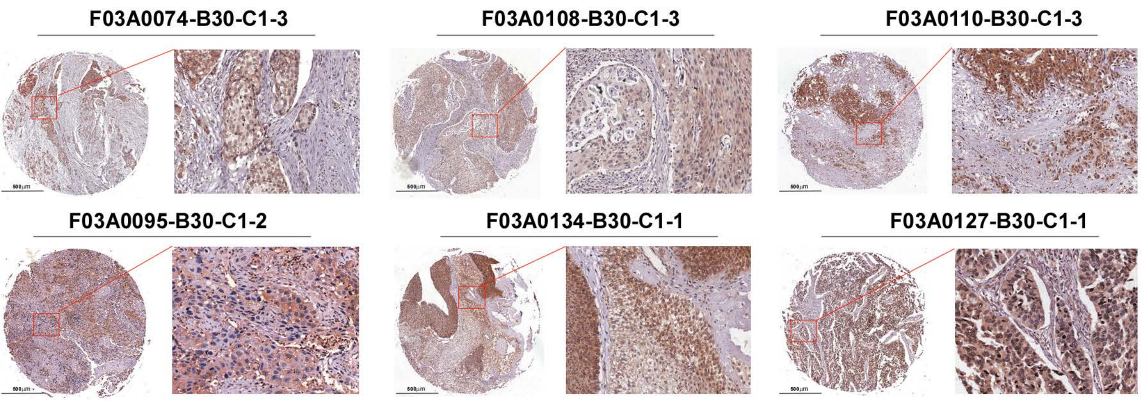

C

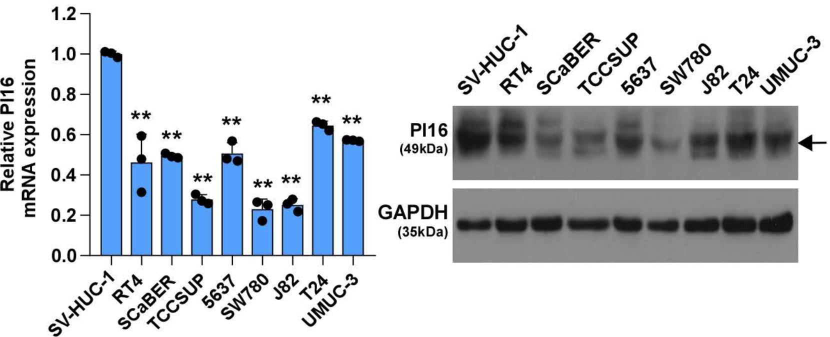

Supplementary Figure 2

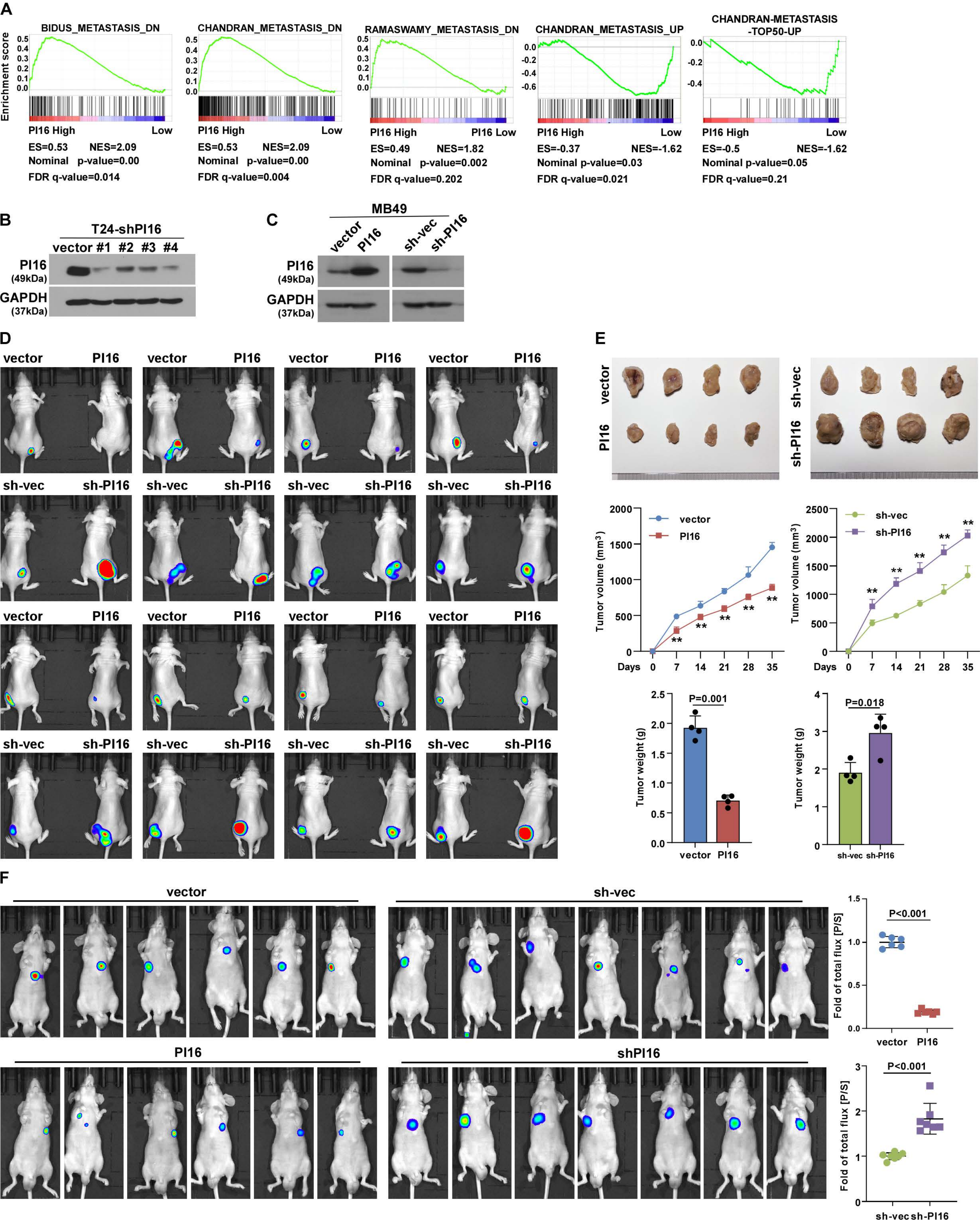

Supplementary Figure 3

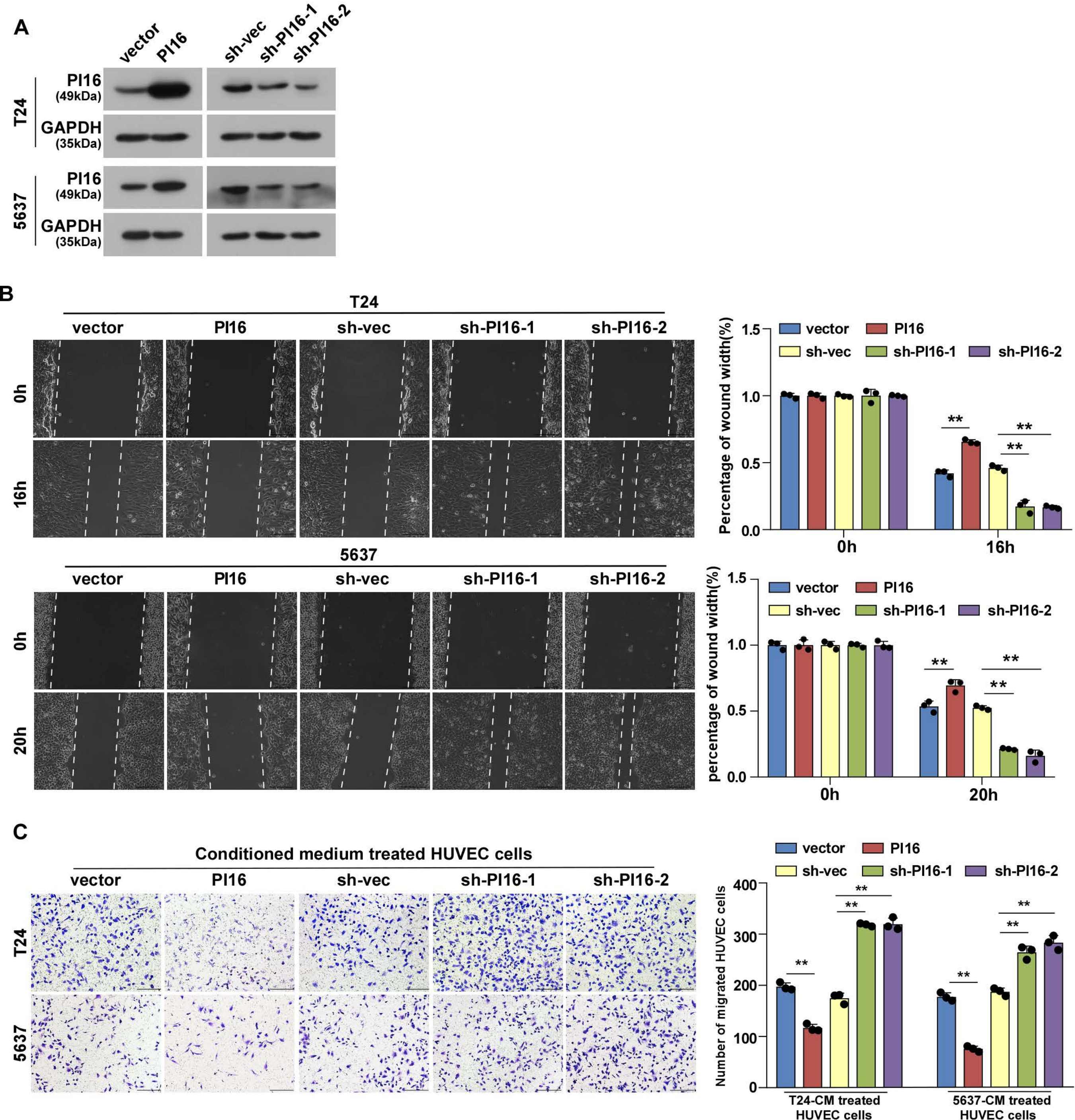

Supplementary Figure 4

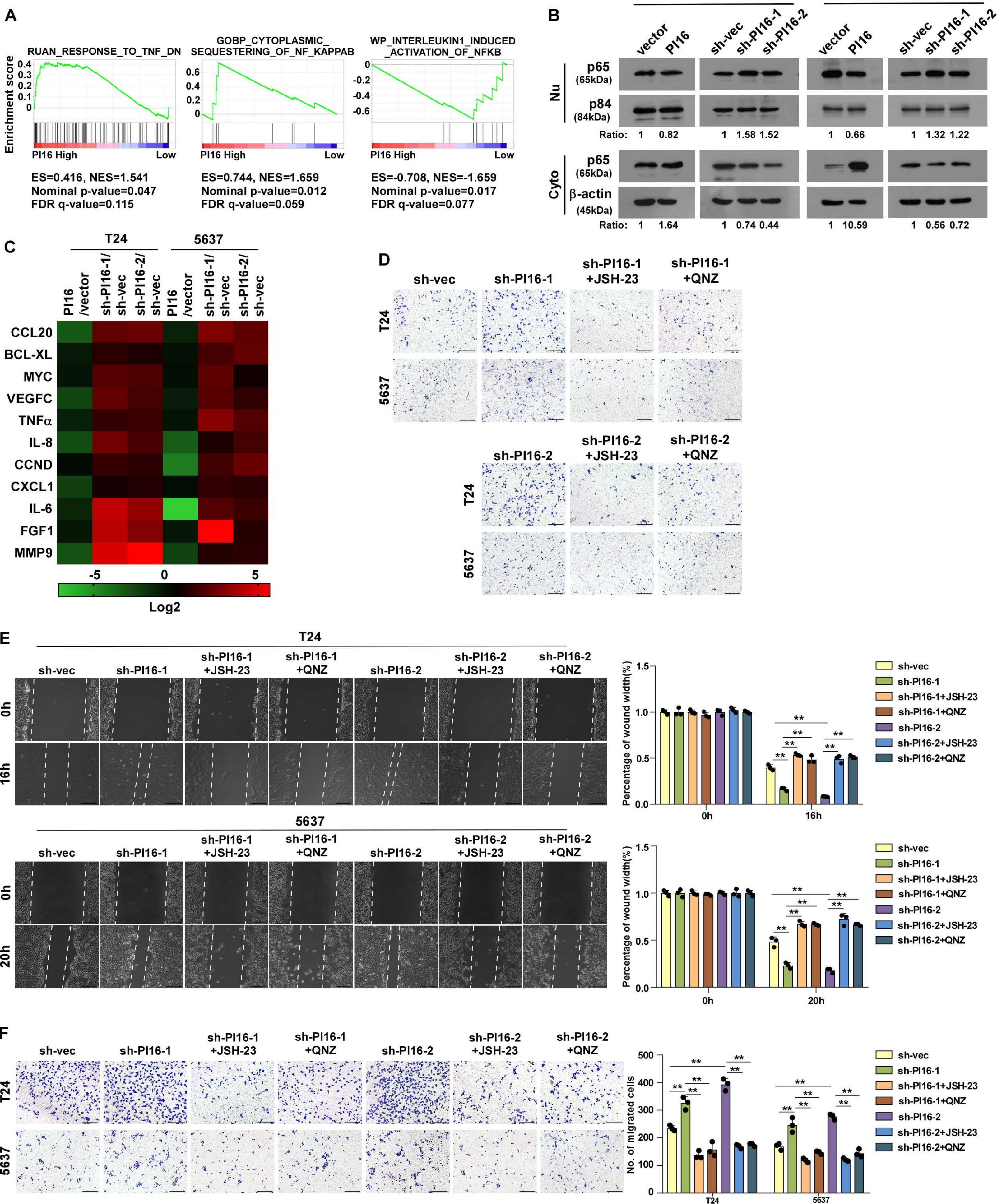

Supplementary Figure 5

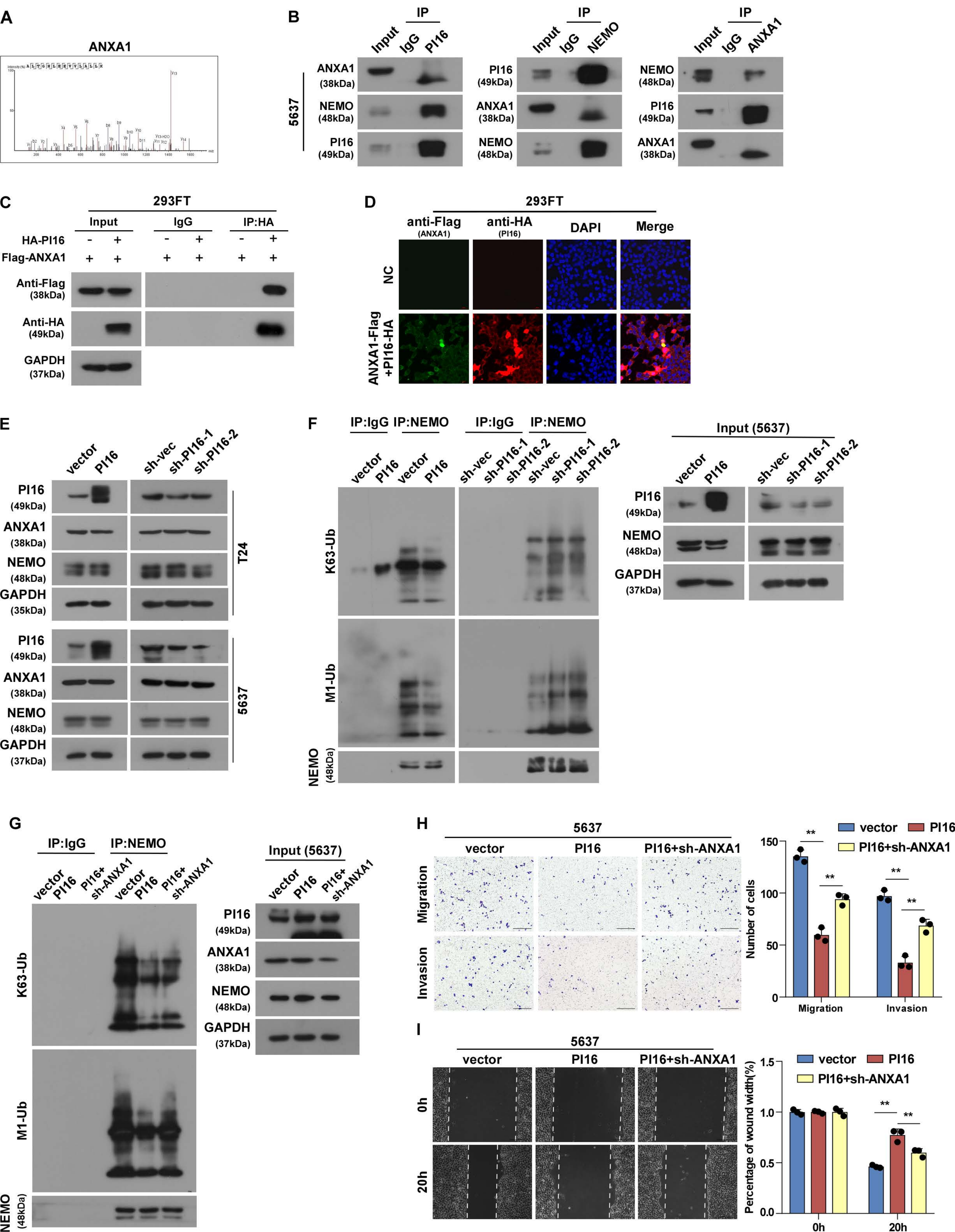

Supplement: Supplementary file 1 — Additional file 1. Supplementary Materials and Methods, Tables and Figures. [file 11658_2023_465_MOESM1_ESM.pdf]
